# Case report: PIK3CA somatic mutation leading to Klippel Trenaunay Syndrome and multiple tumors

**DOI:** 10.3389/fgene.2023.1213283

**Published:** 2023-08-17

**Authors:** Viola Bianca Serio, Maria Palmieri, Simona Innamorato, Lorenzo Loberti, Chiara Fallerini, Francesca Ariani, Enrica Antolini, Jasmine Covarelli, Massimo Vaghi, Elisa Frullanti, Alessandra Renieri, Anna Maria Pinto

**Affiliations:** ^1^ Medical Genetics, University of Siena, Siena, Italy; ^2^ Department of Medical Biotechnologies, Med Biotech Hub and Competence Centre, University of Siena, Siena, Italy; ^3^ Cancer Genomics and Systems Biology Lab, Siena, Italy; ^4^ Genetica Medica, Azienda Ospedaliera Universitaria Senese, Siena, Italy; ^5^ Radiologia Interventistica, Ospedale Maggiore di Crema, Crema, Italy; ^6^ Chirurgia Vascolare, Ospedale Maggiore di Crema, Crema, Italy

**Keywords:** NGS-liquid biopsy, Klippel-Trenaunay Syndrome, squamous cell carcinoma adenocarcinoma, tailored therapy, case report

## Abstract

We report a case of Klippel Trenaunay Syndrome that was monitored both clinically and molecularly over a period of 9 years. A somatic mosaic mutation of *PIK3CA* (p(E545G)) was identified using both cfDNA NGS liquid biopsy and tissue biopsy. At the age of 56, due to intervening clonal mutations in *PIK3CA* background, she developed a squamous cell carcinoma in the right affected leg which was treated surgically. Nine years later, lung bilateral adenocarcinoma arose on *PIK3CA* mutated tissues supported by different clonal mutations. One year later, the patient died from metastases led by a new *FGFR3* clone unresponsive to standard-of-care, immunotherapy-based. Our results highlight the presence of a molecular hallmark underlying neoplastic transformation that occurs upon an angiodysplastic process and support the view that *PIK3CA* mutated tissues must be treated as precancerous lesions. Importantly, they remark the effectiveness of combining cfDNA NGS liquid and tissue biopsies to monitor disease evolution as well as to identify aggressive clones targetable by tailored therapy, which is more efficient than conventional protocols.

## 1 Introduction

Klippel Trenaunay Syndrome (KTS) is a rare congenital disorder characterized by cutaneous vascular malformations involving venous and capillary malformations and presenting cutaneous nevi, limb, bone, and soft tissue hypertrophy (Srividya and Prasanna et al., 2021). It can also present with vascular malformations in the gastrointestinal tract, spleen, genitourinary tract, liver, and heart and can manifest during infancy and progress throughout childhood and adulthood (AlSheef et al., 2020). Visceral involvement in KTS syndrome is not uncommon and presents as Lymphatic malformation of the parenchymal organs or venous infiltration of the hollow organs.

For the diagnosis of KTS, the simultaneous presence of at least two signs between tissue overgrowth, skin nevus, and dilatation of superficial veins is mandatory (Philip and Klippel-Trenaunay, 2019). Regarding the hemodynamic aspects, KTS is a slow-flow malformation in contrast to Parkes-Weber Syndrome, which is a fast-flow malformation. Hemodynamic and anatomic aspects are crucial for the management of KTS. The therapy is usually based on the devascularization of the superficial venous malformation which implies the presence of a patent, normal deep venous system (Lee and Vaghi, 2021). Early genetic studies, aiming to genetically characterize KTS, have shown contradictory data. While, up to date, the majority of these studies reported that KTS patients have no chromosomal defects, there was a study that contradicted these findings, reporting a reciprocal translocation between Chromosomes 5 and 11 or a reciprocal translocation involving chromosomes 8 and 14 (Wang et al., 2001). More recently a link between mosaic-activating mutations of the PIK3CA/mTOR pathway gene and the presence of overgrowth syndromes has been reported (Philip and Klippel-Trenaunay, 2019). Phosphatidylinositol-4,5-bisphosphate3-kinase (PI3K) is one of the crucial kinases in the PI3K/AKT1/mTOR pathway, playing a role in the cellular growth, proliferation, and survival of multiple solid tumors (Hassan et al., 2015). *PIK3CA* gene mutations are reported in several types of cancer, among which breast, colon, endometrium, cerebral, ovarian, lung, head-neck, and skin cancer (Samuels and Todd, 2010). Cutaneous squamous cell carcinoma (CSCC) is an aggressive cancer able to invade and metastasize the dermis and local lymph nodes and several cases are reported where PI3K/AKT/mTOR signaling is hyperactivated and involved in hyperproliferation and tumorigenesis, as well as in apoptosis resistance (Mannella et al., 2021; Mercurio et al., 2021).

cfDNA-NGS Liquid Biopsy (LB) is a non-invasive technique able to detect clonal mutations involved in disease progression that can be used as potential medical targets, if actionable. In this case report, we state the point that combining cfDNA-NGS LB and tissue biopsy, is essential to gain a better understanding of the genetic causes of KTS and solid cancer progression.

We have previously shown that, by comparing genetic data from tissue biopsy and liquid biopsy, we can molecularly characterize vascular malformations (Serio et al., 2022). Here, in addition, we demonstrate a correlation between KTS and CSCC proving that cfDNA-NGS LB is useful for early detection of targetable genetic mutations involved in both diseases.

### 1.1 Diagnostic assessment

#### 1.1.1 Patient enrollment and cfDNA extraction from plasma

The patient underwent genetic counseling and was enrolled at the Medical Genetics Unit of the Azienda Ospedaliera Universitaria Senese, Siena, Italy, for liquid biopsy analysis in order to identify personalized genetic signature that could have explained the patient’s clinic. Written informed consent for genetic analysis was obtained and Clinical information was collected in a genetic consultation setting. Liquid biopsy from both peripheral blood and efferent vein was performed expecting that, as previously demonstrated (Serio et al., 2022), cfDNA would have been more concentrated than the peripheral one. The withdrawal procedure was performed at the Vascular Surgery of Ospedale Maggiore di Crema. The patient was evaluated by a specialist in vascular malformations and underwent a complete radiological workout including duplex scanning and MRI. Different specimens were archived including formalin-fixed paraffin-embedded (FFPE) tissues. Blood samples (10 mL) were collected from the patient and placed into a cell-free DNA BCT^®^ blood collection tube (Streck, Nebraska, United States). Plasma was stored at −80°C until cfDNA extraction. cfDNA was extracted from 4 mL of plasma using MagMAX cell-free Total Nucleic Acid Isolation Kit (ThermoFisher Scientific, Waltham, MA, United States), according to the manufacturer’s instructions. cfDNA quality and quantity were verified respectively using the Agilent™ High Sensitivity DNA Kit (Agilent Technologies, Palo Alto, CA) on Agilent2100 Bioanalyzer (Agilent Technologies) and Qubit™ dsDNA HS Assay Kits on Qubit 3.0 fluorometer (Invitrogen, Carlsbad, CA, United States).

#### 1.1.2 NGS sequencing on cfDNA

cfDNA libraries were prepared using the Oncomine Pan-Cancer Cell-Free Assay (https://www.thermofisher.com/order/catalog/product/A37664). Libraries were loaded on an Ion PI kit using Ion Chef (Thermo Fisher Scientific, Waltham, MA, United States) and sequenced using the Ion Proton sequencer (Life Technologies, Carlsbad, CA, United States). The sequence alignment was performed on the hg19 (human reference genome) using the mapping alignment program (TMAP) with default analysis parameters. The variant calling was performed using Torrent Suite version 5.10.1 (Thermo Fisher Scientific). For the variant annotation Ion Reporter Software 5.20 (Thermo Fisher Scientific) was used. This technology can identify various types of alterations, including single nucleotide variants, insertions/deletions, gene fusions, and copy number variations in cancer-related genes (clinically actionable mutations) with a reportable range up to 0.1%. Its default setting provides several cutoffs for various parameters; in particular, the molecular coverage of at least 3 with a minimum detection cutoff frequency of 0.065% must be satisfied for variant calling of SNV/indel. Regarding the CNV call the MAPD metric, that’s a measure of read coverage noise detected among all amplicons, has to be <0.4, the *p*-value <10–5, and the CNV ratio for a copy number gain >1.15-fold change. All variants called by Variant caller for sensitive detection of variant allele frequency (VAF) up to 0.1%, optimal results are obtained when targeting a median read coverage > 25,000 and a median molecular coverage > 2,500.

#### 1.1.3 Genomic DNA extraction from tissue and sequencing

gDNA was extracted from Formalin-Fixed Paraffin-Embedded (FFPE) using MagCore Genomic DNA FFPE One-Step Kit for MagCore System (Diatech Pharmacogenetics s.r.l., Ancona, Italy) following the manufacturer’s instructions. gDNA was quantified by Qubit Fluorometer with Qubit dsDNA HS Assay (Life Technologies, Carlsbad, CA). Tissue gDNA libraries were prepared with Oncomine Pan-Cancer Cell-Free Assay (https://www.thermofisher.com/order/catalog/product/A37664) and sequenced on ion PI chips using the Ion Proton sequencer (Life Technologies, Carlsbad, CA, United States). The sequence alignment was performed on the hg19 using TMAP with default analysis parameters. The variant calling was performed using Torrent Suite version 5.10.1 (Thermo Fisher Scientific). Ion Reporter Software 5.10 (Thermo Fisher Scientific) was used for the variant annotation.

#### 1.1.4 Genomic DNA extraction from blood and sequencing

Genomic DNA was extracted from EDTA peripheral blood samples using MagCore HF16 (Diatech Lab-Line, Jesi, Ancona, Italy) according to the manufacturer’s instructions. DNA quantity was evaluated using the Qubit 3.0 Fluorometer (Thermo Fisher Scientific, Waltham, MA, United States). The analysis was performed by massively parallel sequencing (Next-Generation Sequencing) on the Proton Ion Torrent platform. Sequencing was preceded by the amplification of the exonic regions of the DNA under investigation, using the Ion AmpliSeq™ Exome Kit.

The qualitative parameters applied that constitute the threshold for the success of the investigation are the following: >90% of reads aligned to the target, >70% of target bases covered at >20X and >40% of target bases covered at >40X. Data analysis was performed using Torrent Suite (v5.0.2)—Life Technologies software, followed by in-house custom patient bioinformatics analysis. Variants considered included: nonsynonymous exonic, splice-site, and frameshift variants not reported in dbSNP142, or reported with unknown or less than 1% frequency in the general population. Synonymous variants, intronic variants not involving splicing sites, and benign variants common in the general population were not considered in the analysis unless previously described as pathogenic. Variants were reported and interpreted in accordance with SIGU and ACMG guidelines (Richards et al., 2007) and the patient’s phenotype as reported by the sending physician.

### 1.2 Case report

A 61-years-old female patient presented to the Medical Genetics Unit of the Azienda Ospedaliera Universitaria Senese in Siena, Italy, for diagnostic purposes in March 2017.

**FIGURE 1:**
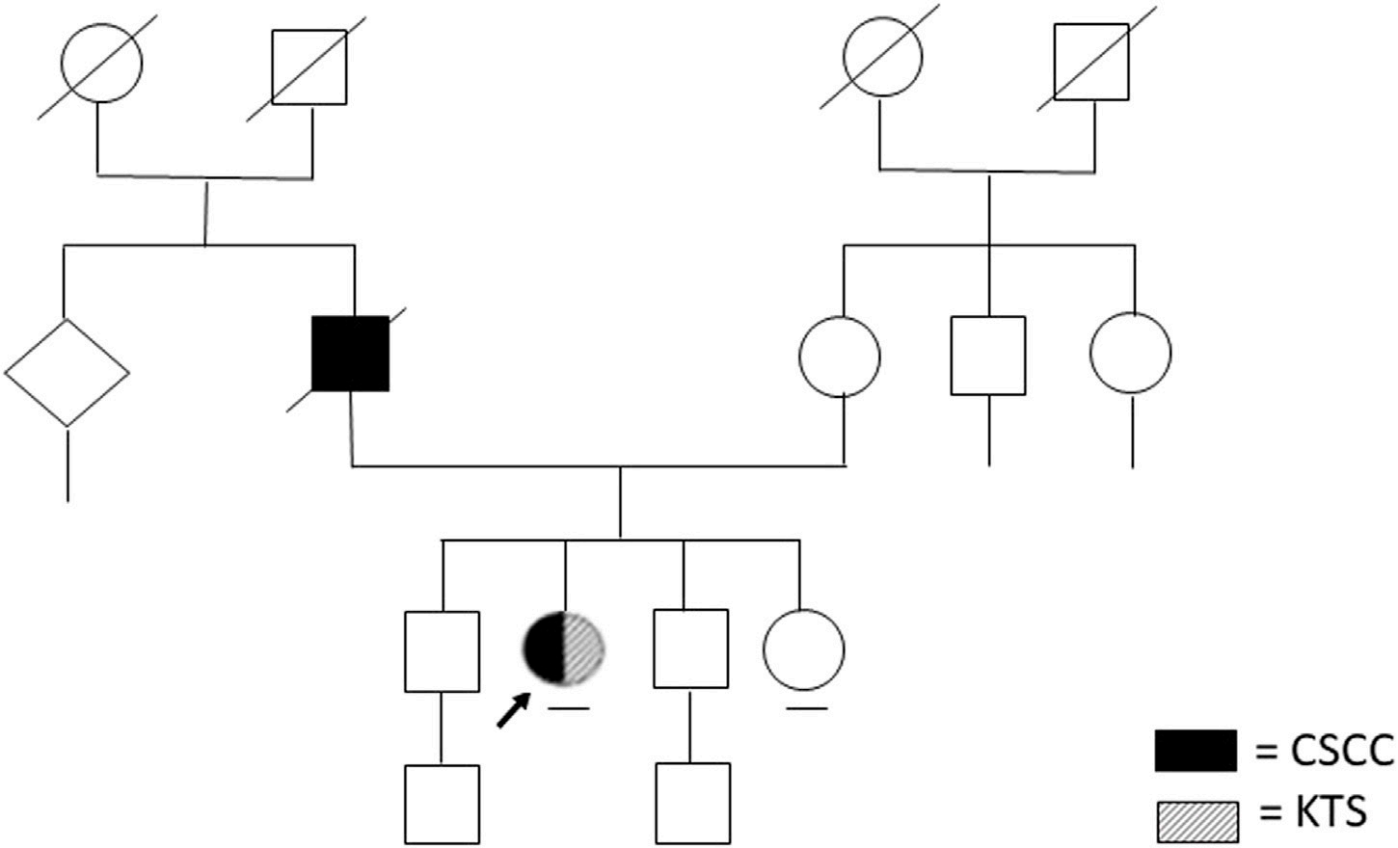
Patient’s family tree.

During the genetic counseling, written informed consent, clinical information as well as the genealogical tree (Figure 1), and cancer family history were collected.

**FIGURE 2:**
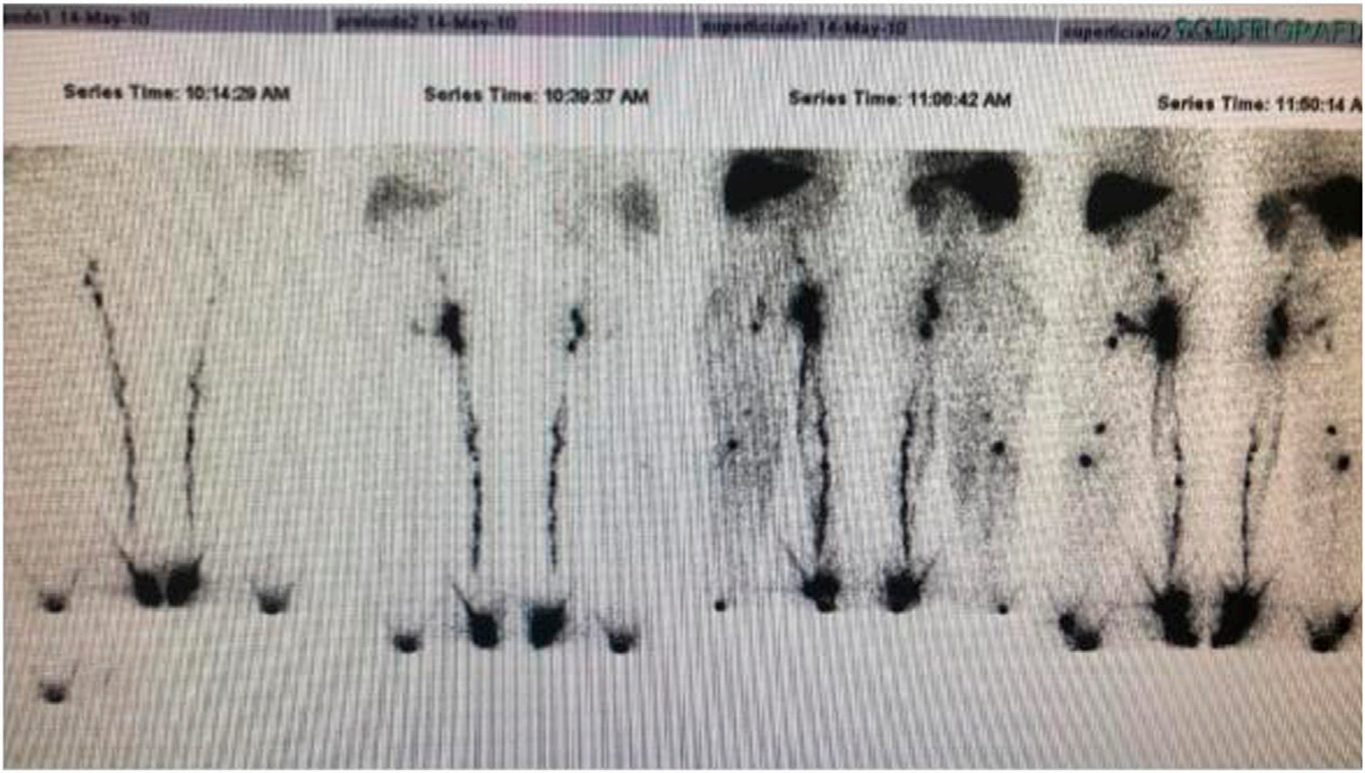
Lymphoscintigraphy: Lack of superficial and deep lymphatics in the right lower limb Lymphatic malformations are often associated to Klippel Trenaunay malformation.

She presented hypertrophy and a congenital angiodysplasia to the lower right limb in the spectrum of Klippel Trenaunay Syndrome (KTS) and chronic infected skin ulcers for which she underwent numerous interventions of sclerotherapy. A lymphoscintigraphy documented a lack of deep and superficial lymphatic vessels (Figure 2).

**FIGURE 3:**
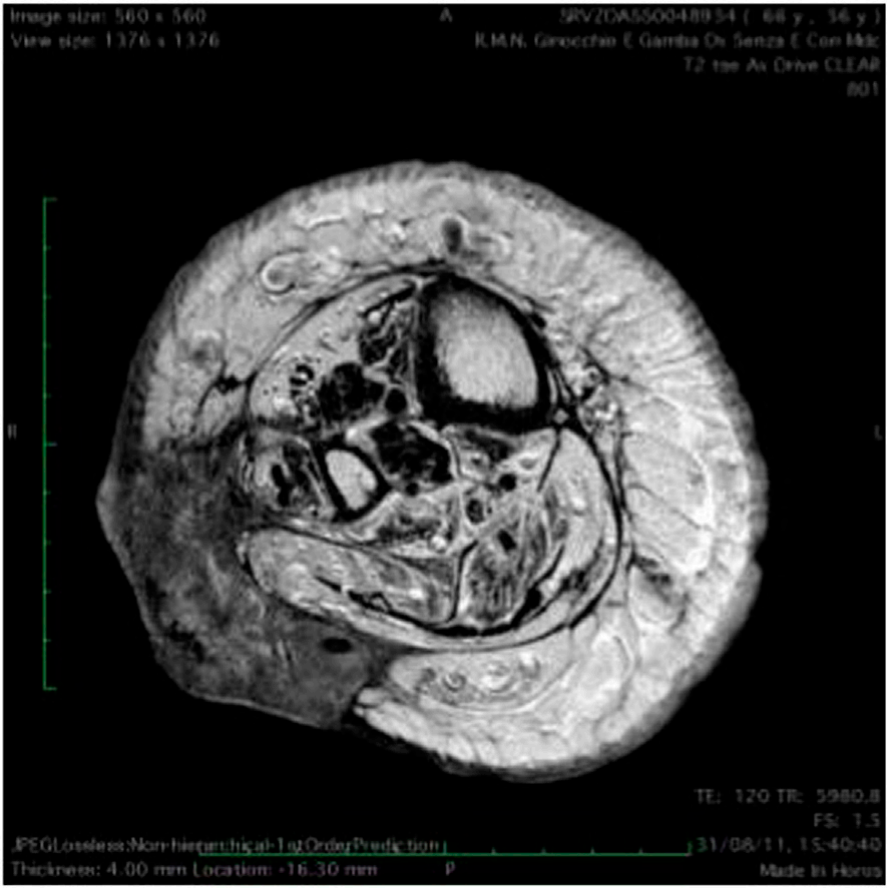
T(1) weighted picture of the leg.

**FIGURE 4:**
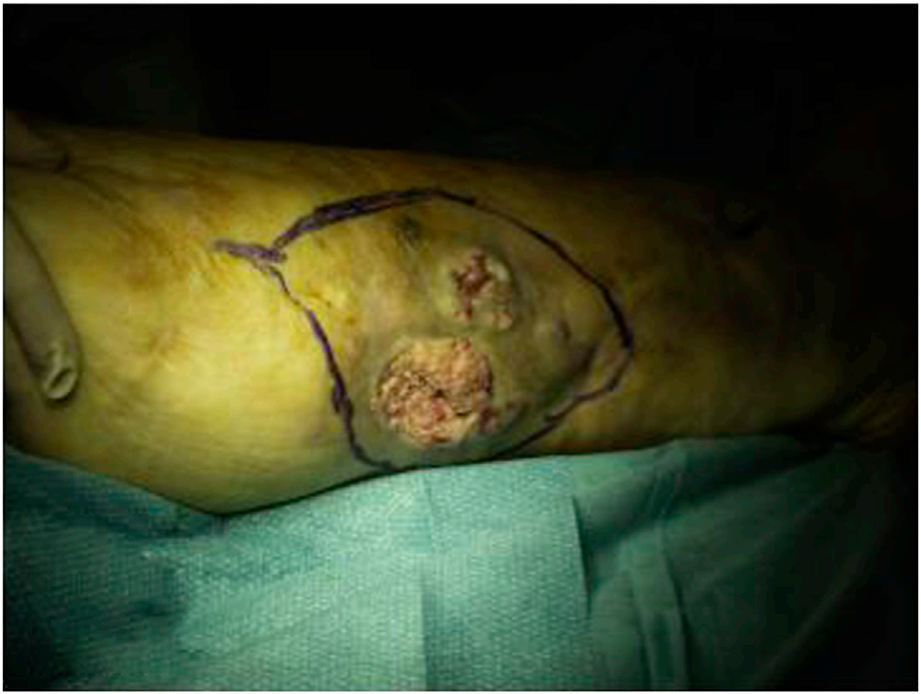
Preoperative picture of the right leg.

**FIGURE 5:**
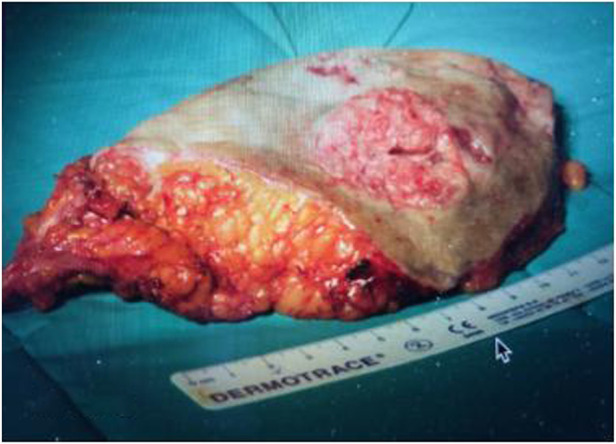
Operatory specimen.

**FIGURE 6:**
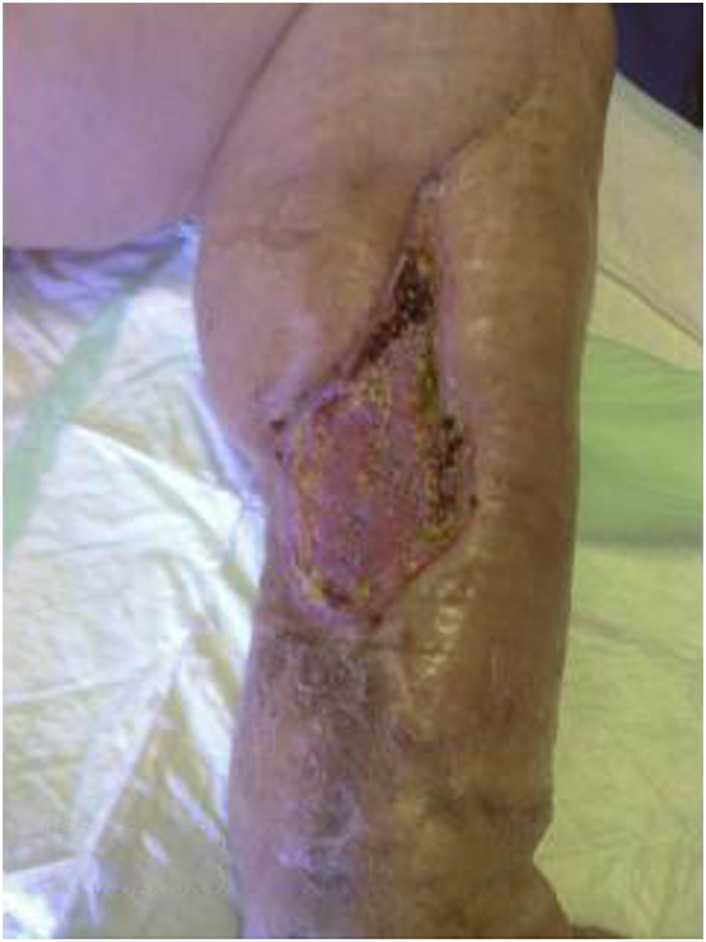
Healing of the surgical wound with skin graft.

At 56 years of age, she had developed a moderately differentiated squamous cell carcinoma at the angiodysplastic lesion site as shown by MRI (Figure 3). The carcinoma had been surgically removed (Figures 4, 5) and coverage of the local flap performed with a skin graft (Figure 6).

**FIGURE 7:**
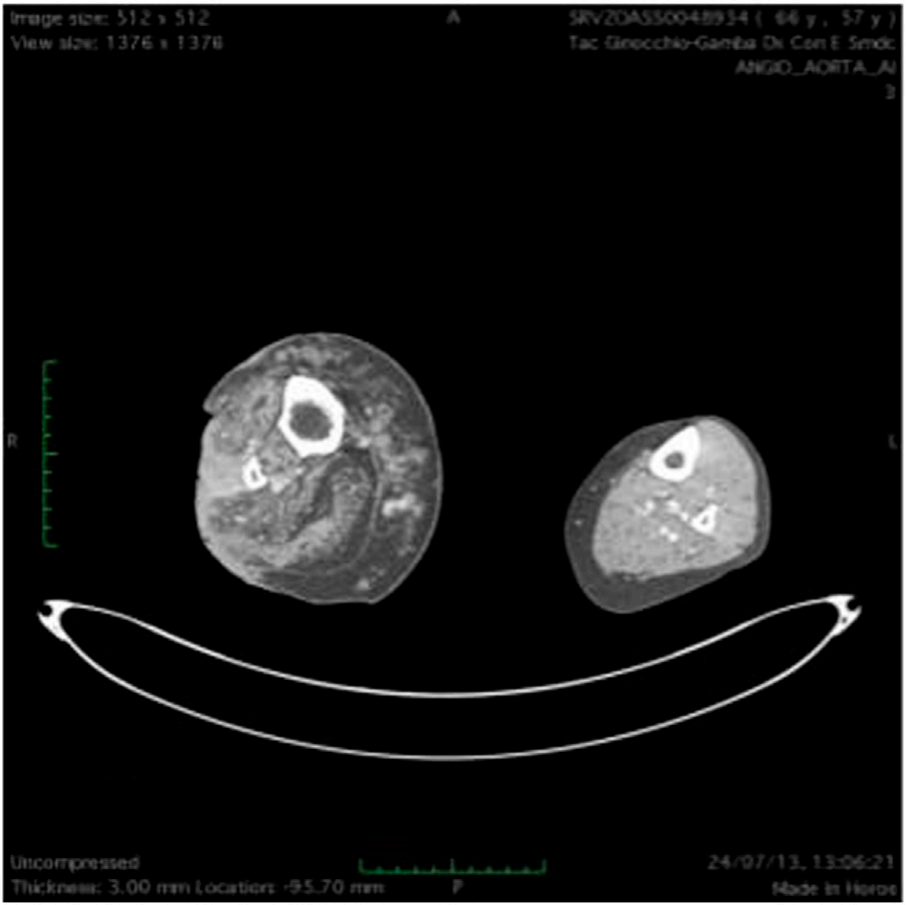
T(2) weighted picture of the lower limbs. Hypertrophy of the soft tissues is present together with dilated ectatic superficial and deep veins. Presence of a neoplastic tissue involving skin and subcutaneous tissue on the peroneal side of the right lower limb.

**FIGURE 8:**
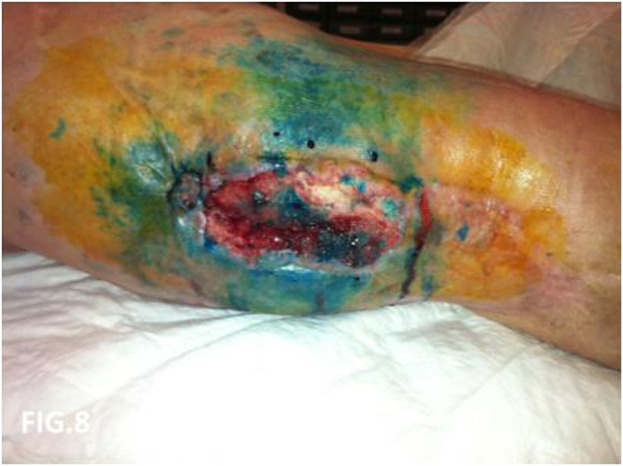
Local recurrence direct phlebography with blue patent. Evidence of dermal backflow.

**FIGURE 9:**
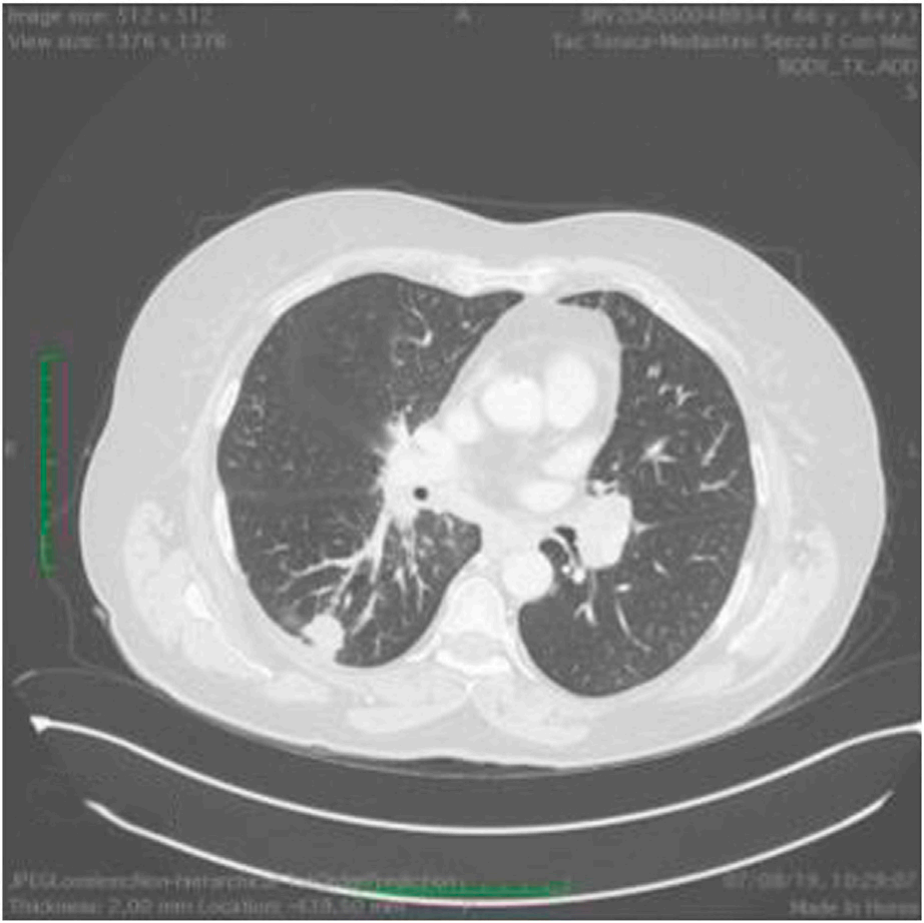
Ct scan demonstrating a mass in the left lung.

**FIGURE 10:**
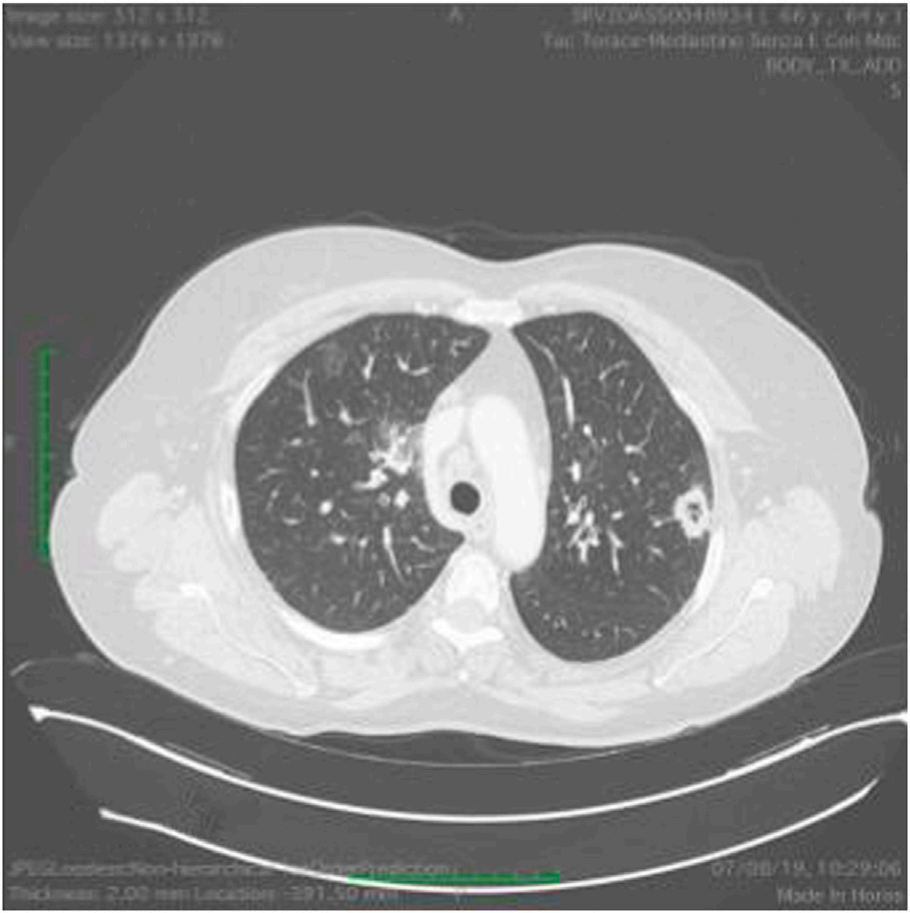
Ct scan demonstrating a mass in the right lung.

At 58 years of age, in October 2013, she had presented a relapse of a well-differentiated and keratinizing squamous cell carcinoma (Figures 7, 8). She consulted with oncologists, and after three attempts to eradicate the lesion that recurred each time, she underwent an amputation up to the middle third of the thigh. The vascular malformation could not be radically resected and a great part remained at the thigh level so that, at the physical examination, there was a visible port-colored spot indicative of residual angiodysplasia. The patient served the interventions of rehabilitation with personalized prostheses. This rehabilitation gave poor results due to the heaviness of the thigh (affected by the congenital vascular syndrome and the high degree of scoliosis and inclination of the pelvis). The sufferer lived in a wheelchair. She was in oncology follow-up with check-ins every day for 6 months. After genetic counseling, in April 2019 the patient developed a superficial vein thrombosis of the stump (she had no thrombosis in the past). In July 2019, when the patient was 63 years old, two lung nodules were discovered. The patient was an active smoker. A bronchoscopy was performed and the analysis of the bronchial aspirate showed the presence of Non-Small-Cell Lung Cancer (NSCLC) as observable in the TC scan sections (Figures 9, 10). Histology discovered the presence of an adenocarcinoma PD-L1 positive.

Anamnestic history was positive for cutaneous squamous cell carcinoma in her father who passed away at 81 years of age (Figure 1).

During genetic counseling, a peripheral blood sample was collected for clinical whole exome sequencing to analyze genetic mutations in genes associated with vascular malformations (Supplementary Material S1). We also obtained angiodysplastic lesion and squamous cell carcinoma tissues from previous surgery (Figure 5) and Oncomine Pancancer analysis was performed on FFPE vascular lesion tissue and squamous cell carcinoma tissue.

During the follow-up in July 2019, peripheral blood samples were collected for cfDNA-NGS liquid biopsy and the patient began immunotherapy with Keytruda (Pembrolizumab). Although the treatment led to a decrease in lung lesions, cerebral metastasis appeared. The further therapy was palliative for overcoming headaches and seizures. Whole Exome Sequencing (WES) from peripheral blood samples identified a constitutive heterozygous mutation in *HGF* c.836G>A, (p.(R279H)). Tissue gDNA analysis, performed on the previous FFPE vascular lesion/squamous cell carcinoma tissue (obtained from the previous surgery, (sample 1- Table 1), detected a clone harboring a *PIK3CA* mutation, c.1634A>G (p.(E545G)) with a VAF of 1,32%.

**TABLE 1:** Patient’s clonal evolution has been monitored using cfDNA-NGS Liquid biopsy over time. In the columns are listed the sample number, the withdrawal date, the biological material and the cfDNA-NGS result with their variant allele frequencies.

Number of sample	Date of withdrawal	Material	cfDNA-NGS result
1	11/2011	FFPE’s vascular lesion + squamous cell carcinoma	*PIK3CA* (p.(E545G))1,3%
2	19/06/2019	Peripheral blood	*PIK3CA*(p.(E545G))0,23%
			*TP53* (p.(G154V)) 0,21%
			0,21%
3	03/09/2019	Lymph Node of adenocarcinoma tissue	*PIK3CA*(p.(E545G))0,10%
4	17/01/2020	Peripheral blood	*FGFR3*(p.(F384L))2,92%
5	17/01/2020	Stump Efferent vein’s withdrawal	*PIK3CA*(p.(E545G))0,078%
			*FGFR3*(p.(F384L)) 41%
			*FGFR3* (p.(Y373C)) 0,23%
			*TP53 *(p.(G154V)) 0,09%

The result from the first peripheral blood sample liquid biopsy (sample 2—Table 1) of the patient, performed after lung cancer discovery, showed the presence of the vascular lesion-related *PIK3CA* mutation, c.1634A>G (p.(E545G)), with a VAF of 0,23%, and of an additional clonal mutation in *TP53* c.461G>T (p.(G154V)) with a VAF of 0,21%.

After 3 months, we performed tissue gDNA analysis on adenocarcinoma’s lymph node tissue (sample 3—Table 1) and it resulted in the detection of the previously identified *PIK3CA* variant, c.1634A>G (p.(E545G)), with a VAF of 0,10%.

In January 2020, we performed two cfDNA-NGS liquid biopsies, one on a new peripheral blood sample and the other on the blood from an efferent vein of the stump.

The liquid biopsy performed on the peripheral blood sample (sample 4—Table 1) identified a new clonal mutation in *FGFR3* c.1150T>C (p.(F384L)), with a VAF of 2,92%.

The liquid biopsy performed on the efferent vein sample confirmed the presence of the mutation in *FGFR3* found in the peripheral blood sample (p.(F384L)) with a VAF of 41%, underlined the persistence of the previously identified *PIK3CA* variant, c.1634A>G (p.(E545G)) with a very low VAF of 0,08% and discovered a new mutation in *FGFR3* c.1118A>G (p.(Y373C)) with a VAF of 0,23%. Various meetings were held with oncologists to introduce *PIK3CA* inhibitors, but the request was rejected due to lacks in the literature.

### 1.3 Discussion

Vascular malformations and malignant tumors share the same angiogenic genes although they display a different clinical evolution. Understanding similarities and differences in the mechanism might contribute to improving therapy of both pathologies.

Indeed in our patient, the use of cfDNA-NGS liquid biopsy allowed us to monitor the KTS scenario associated with the oncologic progression, over time (Supplementary Material S2). Interestingly, we observed the takeover of the different cellular clones responsible for the evolution of the patient’s clinical features. In particular, the exome sequencing on peripheral blood DNA, performed to investigate the genetic cause of the vascular lesion, identified a germline variant of uncertain significance c.836G>A (p.(E545G)) in *HGF*, a gene previously associated with lymphedema (Finegold et al., 2008; Valerie et al., 2015). This variant has not been previously reported in the literature but it is predicted to have a damaging effect on the protein function by bioinformatics tools (CADD_Phred) so we cannot exclude a role in the patient’s clinical phenotype.

The first cfDNA-NGS LB on peripheral blood (sample 2-Table 1) revealed the persistence of the *PIK3CA* pathogenic mutation [c.1634A>G; (p.(E545G)), VAF=0,23%] found in FFPE vascular lesion and in the squamous cell carcinoma tissue obtained from previous surgery (Sample 1-Table 1). The same variant was also present in the metastatic lymph node tissue of the patient at the time of the lung adenocarcinoma although with a lower VAF of 0,10%. Additionally, in the first cfDNA-NGS LB on peripheral blood, there was a pathogenic variant in the *TP53* gene, c.461G>T, (p.(G154V)), with a VAF of 0,21%.

Somatic mutations in *PIK3CA* have been described as being responsible for KTS (Valerie et al., 2015). The identified variant has previously been reported as a PROS causative variant (Siegel et al., 2018) and a clonal driver mutation responsible for the progression of several tumor types, including squamous cell carcinoma and NSCLC (Wang et al., 2014). Therefore, according to the data collected over time on different specimens, in our patient, we can hypothesize a somatic mosaicism for the *PIK3CA* variant which could give reason for both the vascular lesion and the multiple tumors onset and progression. In fact, as it is widely known, the mutations that can be transmitted by inheritance are called “germline”, because they occur in the cells of the germline from which the gametes originate. If, after the union of the gametes, the mutation is transmitted to the new formed zygote, it will be found in every cell of the organism. On the other hand, the mosaicism will be manifest when the genetic error occurs in the early stages of embryo maturation. For this reason the corresponding pathology will be present only in cells derived from that mutated stem cell.

The binding of HGF to its c-MET receptor is responsible for activating the *PIK3CA* pathway and is implicated in the onset of a dysregulated cellular overgrowth as observed in numerous neoplastic processes (Liu et al., 2019; Gesualdi et al., 2020; Xing et al., 2020). Therefore, we cannot exclude that the germline *HGF* identified variant along with the somatic *PIK3CA* mutation may have played a role in the overall patient’s clinical scenario. Additionally, due to her family positive history for cutaneous cancer, the possibility of other germline unidentified mutations contributing to cancer susceptibility cannot be disregarded.

The interest of this case is related to the development of multiple tumors in the patient suffered from a congenital vascular anomaly. In the literature there are few reports on the subject, that is, very interesting and intriguing because the same genes are involved in both pathologies. We need to understand the differences and how best to manage the use of antiangiogenic drugs.

The *PIK3CA* gene is involved in modulating the PIK3/AKT/mTOR pathway and can be a therapeutic target, as well as being associated with standard chemotherapy treatments. Therefore, given the presence of the vascular lesion, the well-defined genetic background of the neoplasia and the poor response to the conventional therapy we discussed with the patient the possibility of asking for a personalized therapy, already approved for other metastatic cancer types such as breast cancer and tailored on her molecular fingerprint. The patient was extremely interested in moving forward with this opportunity; therefore the experimental use of Alpelisib (BYL719) from Novartis was requested. Alpelisib is an inhibitor of the PI3K pathway and its use is approved for the treatment of PIK3CA positive advanced breast cancer. It proved a good efficacy in the treatment of patients suffering from *PIK3CA*-related overgrowth syndrome (PROS) vascular malformations (Quitterie et al., 2018; Juan Carlos et al., 2019). Unfortunately, the ethical committee never approved the treatment of our patient with the *PIK3CA*-inhibitor (Alpelisib) recommending to try all available first-line therapeutic strategies first. The patient underwent immunotherapy with anti-PDL-1 using the drug Keytruda (Pembrolizumab) and, effectively, in January 2020, while we were waiting for approval from the ethical committee, a decrease in lung metastases was observed. Unfortunately, the patient passed away in May 2020 due to the development of cancer metastasis, likely due to a different clonal expansion, despite the ongoing anti-PDL1 treatment.

Notably, in the second peripheral blood liquid biopsy, we observed the presence of a mutation in *FGFR3,* c.1150T>C (p.(F384L)), with VAF of 2,92% while, in the efferent vein of stump-liquid biopsy, we observed the persistence of the *PIK3CA* mutation*,* c.1634A>G (p.(E545G)), with VAF of 0,08% and the *FGFR3* mutation, c.1150T>C (p.(F384L)), with VAF increased up to 41%. Additionally, we observed the co-presence of a second pathogenic mutation in *FGFR3,* c.1124A>G (p.(Y373C)), with VAF of 0,23%. The pathogenic *TP53* (p.(G154V)) clone also reappeared with a VAF of 0,09%. The different clones and related VAFs detected from different withdrawals taken at the same time can be explained by the location of the blood withdrawal. We have previously demonstrated that the total levels of cfDNA in efferent vein withdrawals are higher than in peripheral blood (Serio et al., 2022), so it is likely that in efferent vein withdrawal, we isolated more mutated somatic cells, which explains the increased *FGFR3* VAF and the detection of *PIK3CA* and *TP53* variants, (p.(E545G)) and (p.(G154V)), respectively that were not detected in the peripheral blood. VAF percentage is representative of the number of mutated cells carrying the specific genetic mutation and is inversely related to clinical progression being suggestive of a progressive increase of clonal expansion; increasing VAF rates correlate with greater clinical impact than low rates This evidence, along with the persistence of the clonal mutations in *PIK3CA* and *TP53* genes and the appearance of clonal mutations in the *FGFR3* gene over time, could explain the progression of the patient’s tumor, with the *FGFR3* variants being responsible of the metastatic behavior*.* Furthermore, the *FGFR3* gene is already reported in literature as being a targetable gene in vascular malformations, like Hereditary hemorrhagic telangiectasia (HHT). Tyrosine kinase inhibitor that blocks VEGF receptors like Pazopanib (Votrient) potentially can act as anti-angiogenic treatment in vascular malformations (Aughnan et al., 2019). Although the *FGFR3* gene mutation (p.(F384L)) is annotated as a likely benign variant, we cannot indeed exclude the possibility of an alternative mechanism being involved in cancer progression, considering the upcoming of the pathogenic *FGFR3* subclone (p.(Y373C)). Although there is indeed an overlap between the diagnostic assessment section presented in this manuscript and the one published in the previous work (Serio et al., 2022), the purpose of the current manuscript goes beyond the purpose of the previous paper. The present manuscript does not mainly focus on the powerful use of liquid biopsy in the diagnosis of vascular malformations but it aims to highlight the connection between the dysregulated cellular overgrowth observed in angiodysplastic processes and the one observed in carcinogenesis. This one presents an overall amount of additional molecular and clinical data to unveil the molecular background underlying the oncological transformation that occurs upon KTS lesions. Our patient indeed was not only affected by KTS syndrome, but also presented with neoplastic involvement; the employment of liquid biopsy allowed us to identify the molecular hallmark that could explain the combination of both pathologies.

### 1.4 Conclusion

This manuscript firstly unveils the molecular background underlying the oncological transformation that occurs upon an angiodysplastic process.

Liquid biopsy fulfills the need to monitor the dynamic evolution of malignancies helping to choose the best target therapy according to the genetic scenario. This case report highlights the novelty of using liquid biopsy as a tool to explore both the somatic part of the tumor and the mosaicism of KTS pathology with a single test. Again, it stresses the possibility of detecting potential therapeutic targets in a timely manner. It also underlines the need to promptly access experimental treatments that may be more effective than standard protocols.

The patient we described represents a link between the vascular malformations world and malignant tumors. Vascular malformations represent a static genetic process since birth whereas malignancies are very dynamic. Although our work could suggest a higher risk for neoplastic disease in angiodysplastic malformation affected patients further studies are needed to better dissect the functions of each gene and the effects of the functional relationships.

## Data Availability

The data presented in the study are deposited in the SRA repository, accession number PRJNA1000753.

## Supplementary material

Supplementary Figure S1 List of patient’s analyzed samples. The detected mutations are reported for each sample.
