# Supplementary figures and images for: Case report: PIK3CA somatic mutation leading to Klippel Trenaunay Syndrome and multiple tumors
Source: Front Genet. 2023 Aug 17;14:1213283. doi: 10.3389/fgene.2023.1213283 (PMC10469863; doi:10.3389/fgene.2023.1213283)

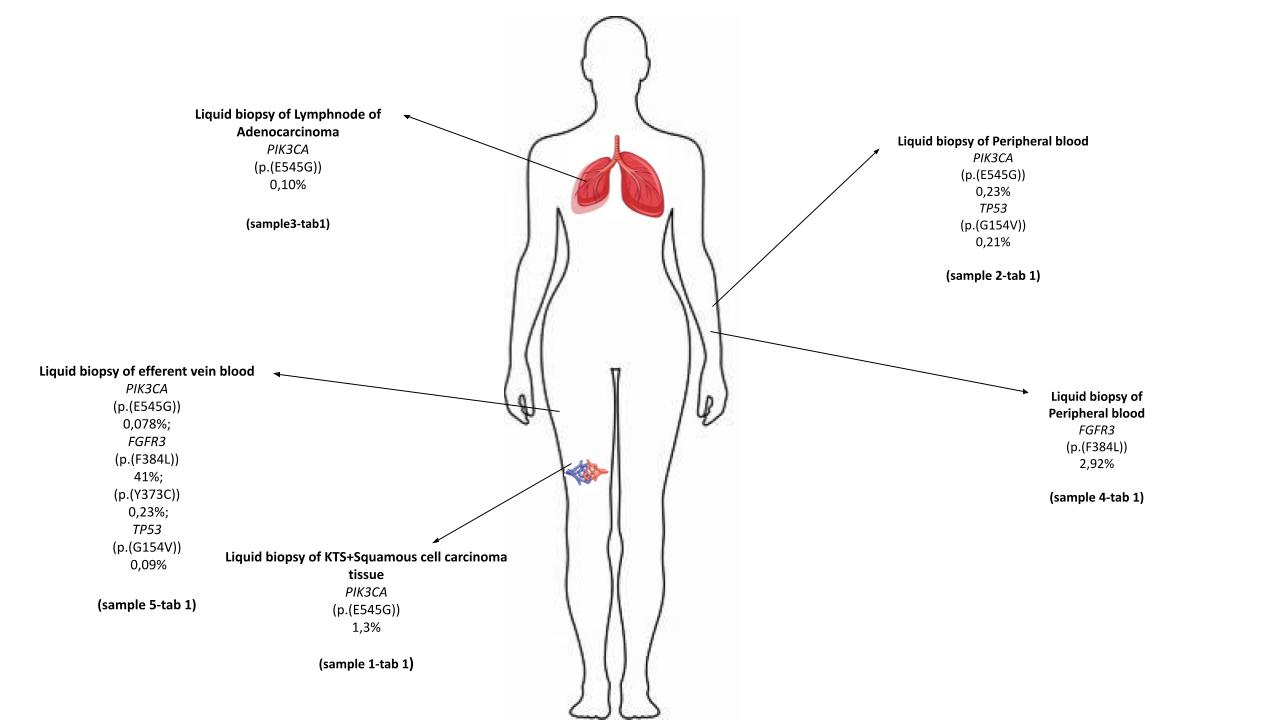

Supplement: Supplementary file 1 [file Image1.JPEG]
