# Supplementary material for: Case report: PIK3CA somatic mutation leading to Klippel Trenaunay Syndrome and multiple tumors
Source: Front Genet. 2023 Aug 17;14:1213283. doi: 10.3389/fgene.2023.1213283 (PMC10469863; doi:10.3389/fgene.2023.1213283)
Supplement: Supplementary file 2 [file DataSheet1.docx]

***Vascular genes panel***

*GNA11* (OMIM #139313; Ref Seq NM_002067.4; Assembly GRCh37/hg19),

*GNAQ* (OMIM #60998; Ref Seq NM_002072.4; Assembly GRCh37/hg19),

*AKT1* (OMIM #164730; Ref Seq NM_005163.2; Assembly GRCh37/hg19),

*KRAS* (OMIM #190070; Ref Seq NM_033360.3; Assembly GRCh37/hg19),

*HGF (*OMIM *#142409; Ref Seq NM_000601.6; Assembly Grch377hg19)*,

*HRAS* (OMIM #190020; Ref Seq NM_005343.3; Assembly GRCh37/hg19),

*IDH1* (OMIM #147700; Ref Seq NM_005896.3; Assembly GRCh37/hg19),

*PIK3CA* (OMIM #171834; Ref Seq NM_006218.3; Assembly GRCh37/hg19),

*TEK* (OMIM #60221; Ref Seq NM_000459.4; Assembly GRCh37/hg19),

*PTEN* (OMIM #601728; Ref Seq NM_000314.7; Assembly GRCh37/hg19),

*RASA1* (OMIM #139150; Ref Seq NM_002890.2; Assembly GRCh37/hg19),

*TGFBR2* (OMIM #190182; Ref Seq NM_001024847.2; Assembly GRCh37/hg19),

*TGFBR1* (OMIM #190181; Ref Seq NM_004612.3; Assembly GRCh37/hg19),

*SMAD3* (OMIM #603109; Ref Seq NM_005902.3; Assembly GRCh37/hg19),

*SMAD4* (OMIM #600993; Ref Seq NM_005359.5; Assembly GRCh37/hg19),

*TGFB1* (OMIM #190180; Ref Seq NM_000660.6; Assembly GRCh37/hg19),

*TGFB2* (OMIM #190220; Ref Seq NM_001135599.3; Assembly GRCh37/hg19),

*GLMN* (OMIM #601749; Ref Seq NM_053274.2; Assembly GRCh37/hg19).
